# Extension of the Lecture-Term

**Published:** 1849-01

**Authors:** 


EXTENSION OF THE LECTURE-TERM.

Prof. Joseph A. Eve states in his Introductory Lecture, that the Medical College of Georgia at its organization, adopted six months as the length of their lecture-term, but that after persevering five years "with little encouragement and patronage," the professors finally came down to the short sessions of other schools. "After the adoption of the four month's term," he remarks, "their classes increased rapidly."

We take great pleasure in quoting this piece of medical history, not only as an act of justice to a highly respectable institution, but as illustrative of that progress in the profession signs of which are visible all around us. A number of schools have adopted a lengthened term, and they experience no diminution in the size of their classes. A few years have wrought a signal change in professional opinion, and the schools are conforming to that enlightened judgment. In all the schools the sessions, in a few years, will be extended; and dissections and clinical instruction will be insisted upon, as pre-requisites to graduation. It gratifies us to learn that the Medical College of Georgia is prosperous. With a gifted faculty, alive to the true glory of their profession, ready to second every movement tending to its advancement, high-toned and manly, it deserves to prosper.

